# Mesenchymal stem cells promote osteosarcoma cell survival and drug resistance through activation of STAT3

**DOI:** 10.18632/oncotarget.10219

**Published:** 2016-06-22

**Authors:** Bing Tu, Jing Zhu, Shen Liu, Lei Wang, Qiming Fan, Yongqiang Hao, Cunyi Fan, Ting-Ting Tang

**Affiliations:** ^1^ Shanghai Key Laboratory of Orthopedic Implants, Department of Orthopedic Surgery, Shanghai Ninth People's Hospital, Shanghai Jiao Tong University School of Medicine, Shanghai, 200011, China; ^2^ Department of Orthopedic Surgery, Shanghai Jiao Tong University Affiliated Sixth People's Hospital, Shanghai, 200233, China; ^3^ Department of Integrative Medicine and Neurobiology, Fudan University, Shanghai 200032, China

**Keywords:** osteosarcoma, mesenchymal stem cells, chemotherapy, STAT3

## Abstract

Increasing evidence suggests that the tumor microenvironment plays a key role in the development of drug resistant tumor cells. In this study, we tried to determine whether the mesenchymal stem cells (MSCs) in the tumor microenvironment contribute to the increased chemoresistance of osteosarcoma. We found that exposure of Saos-2 and U2-OS cells to MSCs conditioned medium (CM) increased the viable cells in the presence of therapeutic concentrations of doxorubicin or cisplatin. Meanwhile, the MSC CM-associated pro-proliferative effects were accompanied by reduced caspase 3/7 activity and Annexin V binding. We confirmed that STAT3 activation by IL-6 regulates MSCs-induced chemoresistance. Blockade of this signal re-sensitized drug-resistant Saos-2 cells to drug treatment. Using a osteosarcoma mouse model with co-injection of MSCs with Saos-2cells, we found that inhibition of STAT3 prolonged the survival time of tumor bearing mice by suppressing tumor growth and increasing the sensitivity of tumor cells to doxorubicin. Finally, we demonstrated that increased expression of p-STAT3, multidrug resistance protein (MRP) and P-glycoprotein (MDR-1) was associated with high chemotherapy resistance in clinical osteosarcoma samples. Collectively, our findings suggest that MSCs within the tumor microenvironment may represent a new target to enhance chemotherapeutic efficacy in osteosarcoma patients.

## INTRODUCTION

Osteosarcoma is the most common primary malignant tumor in bone and accounts for approximately 5% of all newly diagnosed pediatric cancers [1]. Currently, patients with osteosarcoma receive approximately 10 weeks of chemotherapy, followed by surgical resection of the primary tumor [2]. The standard protocol of care for treating primary and metastatic osteosarcoma is a three-drug chemotherapy regimen consisting of cisplatin, doxorubicin and methotrexate [3, 4]. Although higher survival rates have been achieved with combined chemotherapy, unfortunately, osteosarcoma is a relatively drug resistant disease, and the question of when chemotherapy resistance emerges in osteosarcoma is still unknown [5]. Therefore, understanding the development of resistance in these patients will be beneficial for a large group of patients.

Although previous studies have identified various tumor cell-intrinsic mechanisms of drug resistance, it has become increasingly clear that the tumor microenvironment plays a key role in the development of drug resistance [6, 7]. Consequently, researchers have demonstrated that stromal cells in the microenvironment are important for modulating a chemotherapy response and are often an indicator of poor prognosis [8]. For example, it has been reported that MSCs can support tissue regeneration in response to therapy [9, 10]. MSCs are found predominantly in the bone marrow, a common origin site for osteosarcoma. In the tumor, MSCs are found to promote the growth, angiogenesis and metastasis of tumor cells through the release of a spectrum of cytokines [11, 12].

Increasing evidence supports the idea that interactions between MSCs and tumor cells plays a critical role in the initiation and development of carcinomas [13, 14]. However, the majority of reports have focused on proliferative, angiogenic, and immune-regulation effects [15, 16]. Our previous studies have identified a pro-proliferative and metastatic effect of human MSCs on osteosarcoma cells in the bone marrow microenvironment [17, 18]. A later study identified mesenchymal stem cell-secreted IL-6 as a critical mediator of this interaction [19]. Based on these observations, we hypothesized that MSCs may also induce chemotherapy resistance and promote the failure of current chemotherapies in osteosarcoma patients. In this study, we investigated the effect of MSCs on chemotherapy resistance in two osteosarcoma cell lines, Saos-2 and U2-OS, *in vitro* and *in vivo*.

## RESULTS

### MSCs protect osteosarcoma cells from drug-induced apoptosis

**Figure 1:**
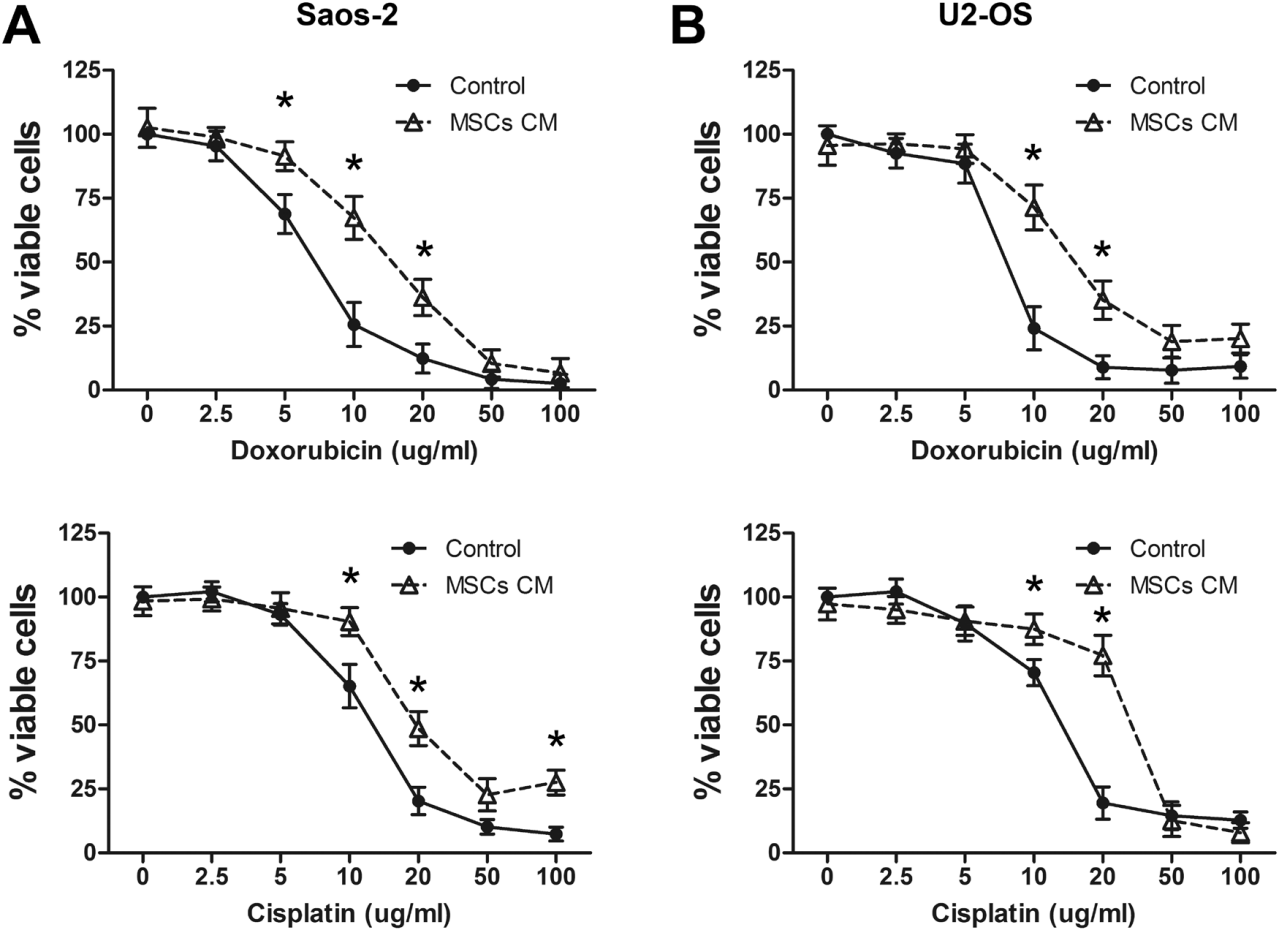
MSCs promote survival in drug-treated osteosarcoma cells **A.** Saos-2 cells (1×10^4^) were cultured in the presence or absence of MSCs CM containing 1% FBS and treated with doxorubicin (top) or cisplatin (bottom) for 4 days. The number of viable cells was tested by the CCK-8 assay. The data represent the mean±SD percentage of viable cells for indicated drug concentrations. **B.** Cell viability of U2-OS cells (1×10^4^) that were cultured and treated as Saos-2 for 4 days. The data points represent the mean cell number from 3 independent experiments, and the error bars represent SD.

Human MSCs harvested from the bone marrow were characterized by both surface marker stainings and functional studies (Supplemental Figure S1A, S1B). We then investigated whether MSCs also protect osteosarcoma cells from drug-induced apoptosis. Our study focused on doxorubicin and cisplatin, which are two of the most commonly used drugs for osteosarcoma chemotherapy [20]. Saos-2 and U2-OS osteosarcoma cells were exposed to different concentrations of chemotherapy drugs in the presence or absence of MSCs CM containing 1% FBS for 24 h. Saos-2 cells that were grown in MSCs CM and treated with 10 or 20 μg/ml doxorubicin or cisplatin (concentrations ranging from 0 to 100 μg/ml) showed a significant increase in survival compared with cells cultured in regular medium (control; Figure 1A). Similarly, U2-OS cells treated with 10 or 20 μg/ml doxorubicin or cisplatin displayed significantly greater survival when cultured in the presence of MSCs CM compared to regular medium (Figure 1B).

**Figure 2:**
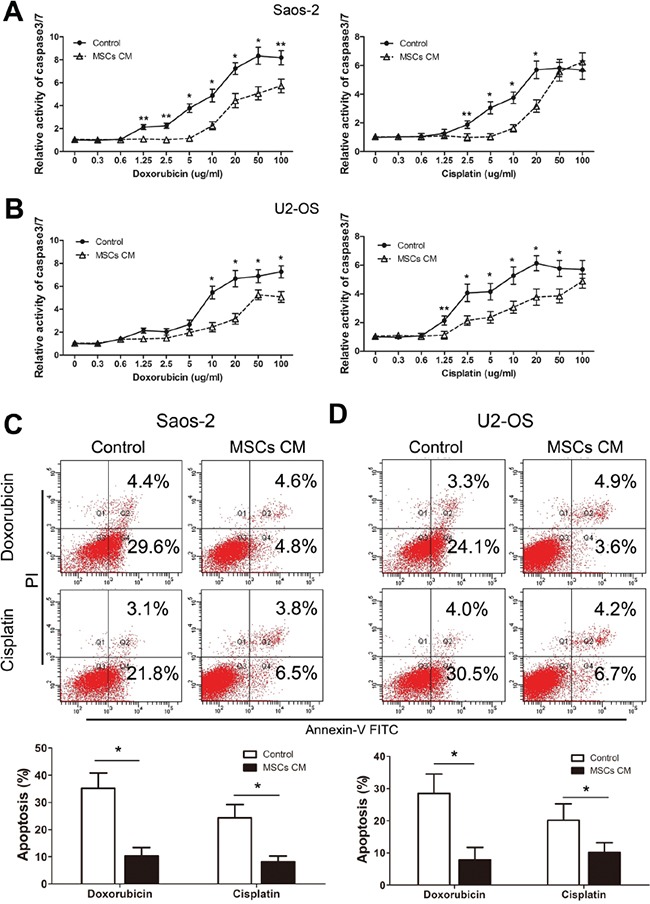
MSCs protect osteosarcoma cells from drug-induced apoptosis **A.** Saos-2 cells (1×10^4^) that were cultured in the presence or absence of 1% serum MSCs CM and treated with doxorubicin (left) or cisplatin (right) at indicated concentrations for 48 hours were examined for caspase 3/7 activity. The data represent the mean±SD fold change from Saos-2 cells cultured in DMEM (Control) in triplicate wells. **B.** U2-OS cells (1×10^4^) were cultured and treated as Saos-2 were examined for caspase-3/7 activity. **C.** Saos-2 cells that were cultured in the presence or absence of 1% serum MSCs CM and treated with doxorubicin or cisplatin (20 μg/ml) for 48 hours were examined for Annexin V binding using FACS analysis. Top, representative analysis by FACS at 48 hours. Bottom, data represent the mean±SD of Annexin V–FITC positive cells of three independent experiments. **D.** U2-OS cells (1×10^4^) were cultured and treated as described for Saos-2 cells, and FACs analysis was used to assess the percentage of apoptotic cells. n=3; *, p<0.01; ** p<0.05.

Consistent with the protective effect of MSCs, we observed a significantly lower level of caspase 3/7 activity in Saos-2 cells that were cultured in the presence of MSCs CM, compared to control medium and exposed to doxorubicin (2.5 to 100 μg/ml) or cisplatin (2.5 to 20 μg/ml, Figure 2A). U2-OS cells treated with MSCs CM also displayed significantly lower levels of caspase 3/7 activity than cells cultured with control media in the presence of doxorubicin or cisplatin (Figure 2B). Additionally, the protective effect of MSCs CM on drug-induced tumor cell apoptosis was confirmed by flow cytometry analysis. Saos-2 cells cultured in MSCs CM or control medium containing 1% FBS were exposed to doxorubicin or cisplatin for 48 h and then stained with Annexin V–FITC and PI. Our data indicated a significant reduction in the percentage of apoptotic cells in drug-treated Saos-2 cells that were exposed to MSCs CM (Figure 2C). Meanwhile, culturing tumor cells in 1% FBS had no significant effect on the proliferation and apoptosis (Supplementary Figure S2A and S2B). U2-OS cells that were exposed to MSCs CM also showed a significant reduction in apoptosis at 48 h (Figure 2D). Our data demonstrate that MSCs exert a pro-survival effect on osteosarcoma cells by protecting them from drug-induced apoptosis. Furthermore, previous studies have reported that behavior of MSCs are tissue independent. We then compared the bone marrow-derived MSCs with umbilical cord derived MSCs. Our results showed that both MSCs had similar effects on the drug-induced apoptosis (Supplementary Figure S3).

### STAT3 is essential for MSC-induced chemoresistance in osteosarcoma cells

**Figure 3:**
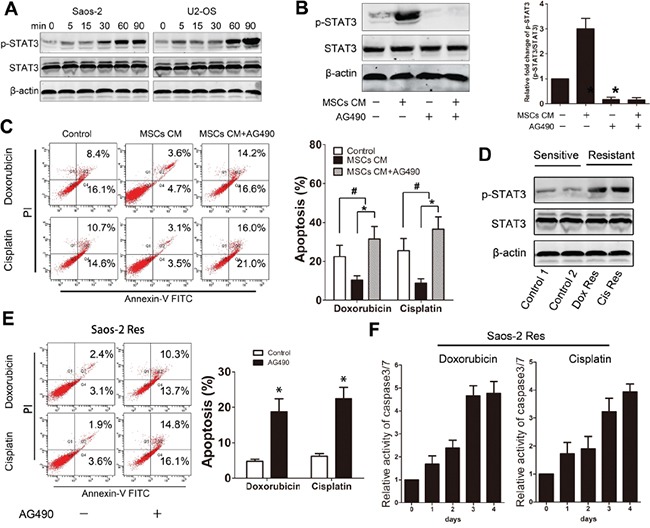
STAT3 is essential for MSC- and drug-induced osteosarcoma chemoresistance **A.** Saos-2 and U2-OS cells were treated with MSCs CM for the indicated times, and p-STAT3 and STAT3 levels were examined by Western Blot. **B.** Saos-2 cells were cultured in the presence or absence of MSCs CM and treated with AG490 for 30 min. Left, p-STAT3 and STAT3 levels were examined by Western Blot. Right, the data represent the means ± SD fold change from Saos-2 cells cultured in DMEM (Control) in three independent experiments. **C.** Saos-2 cells were cultured in MSCs CM in the presence or absence of AG490 for 48 hours. Left, Annexin V binding was tested by FACS. Right, data represent the mean ± SD of apoptotic cells of three independent experiments. **D.** Chemoresistant Saos-2 cells were obtained from low dose exposures to (1 μg/ml) doxorubicin or cisplatin treatment. p-STAT3 and STAT3 levels were assessed by Western blot. **E.** Chemoresistant Saos-2 cells were exposed to corresponding drugs (20 μg/ml) for 48 hours. Left, Annexin V binding was tested by FACS. Right, data represent the mean±SD of apoptosis cells of three independent experiments. **F.** Chemoresistant Saos-2 cells that were treated with AG490 were exposed to corresponding drugs (20 μg/ml) for 4 days. Caspase 3/7 activity was tested at the indicated times. n=3; *, p<0.01; #, p>0.05.

Activated STAT3 signaling has been shown to regulate chemoresistance in various tumors. Our previous research indicated that STAT3 is a critical mediator for osteosarcoma proliferation [19]. To determine whether STAT3 signaling is involved in MSC-induced osteosarcoma survival, we assessed whether activated STAT3 levels were increased in response to MSC-CM treatment. Activation of STAT3 was observed in both osteosarcoma cell lines 30 minutes after incubation with MSCs CM (Figure 3A). Treatment of Saos-2 cells with AG490 (JAK2 inhibitor) inhibited STAT3 activation. However, the inhibitory effect of AG490 on STAT3 could not be re-activated by treatment with MSC-CM (Figure 3B). Next, Saos-2 cells that were cultured in MSCs in the presence or absence of 50 μM AG490 were treated with 20 μg/ml doxorubicin or cisplatin for 48 h. Inhibition of STAT3 significantly enhanced the drug-induced apoptosis of the tumor cells (Figure 3C). To obtain drug resistant osteosarcoma cells, Saos-2 cells were cultured in a low dose of doxorubicin or cisplatin (1 μg/ml) and passaged every 3 days. The surviving Saos-2 cells were regarded as chemoresistant tumor cells. The STAT3 activation levels in the cells were tested by western blot. We observed an increase in STAT3 activation levels in both doxorubicin- and cisplatin-resistant cells (Figure 3D). Chemoresistance was confirmed by exposing cells to 20 μg/ml doxorubicin or cisplatin for 24 h followed by FACS analysis, which revealed few apoptotic cells. However, the inhibition of STAT3 via AG490 treatment in the chemoresistant cells led to a significant increase in the apoptosis rate (Figure 3E). Finally, the chemoresistant Saos-2 cells were treated with drugs in the presence of AG490 for 4 days. We observed that AG490 treatment increased the caspase 3/7 activity in the chemoresistant tumor cells (Figure 3F). Together, these results suggest that STAT3 modulates the protective effects of MSCs on osteosarcoma cells. Moreover, inhibition of STAT3 may enhance chemotherapeutic sensitivity in cases of MSCs- or drug-induced resistance.

### MSCs promotes the proliferation and survival of osteosarcoma *in vivo*

**Figure 4:**
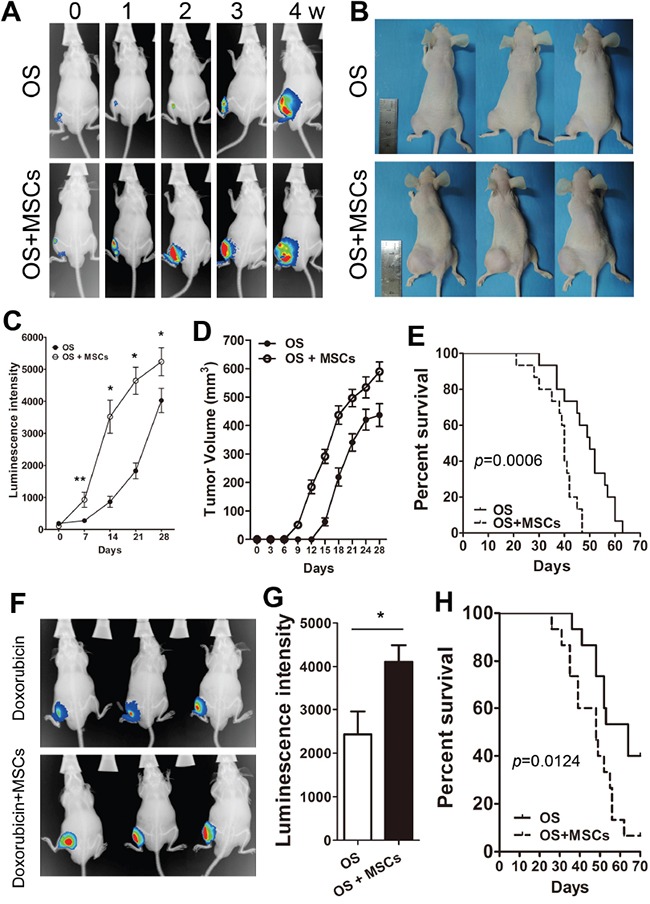
MSCs suppress drug-induced tumor regression *in vivo* **A.** Saos-2 cells (5 *10^6^) were injected in the left tibia of Balb/c nude mice in the absence (OS) or presence (OS+MSCs) of 5 *10^6^ MSCs. The mice were maintained for 4 weeks and serial bioluminescent imaging of mice was obtained at indicated times. **B.** The representative gross tumor volume images of three mice at week 4. **C.** The weekly quantification of luminescent signals from tumor bearing mice after Saos-2/MSCs injection. **D.** The quantification of tumor volumes at the indicated times. **E.** The overall survival of mice bearing Saos-2 or Saos2/MSCs. (log-rank test, n = 12, p = 0.0006). **F.** The OS and OS+MSCs group of mice were treated with doxorubicin (10 mg/kg) via weekly intraperitoneal injections and *in vivo* images of the OS and OS+MSCs group mice are shown at week 4. **G.** Quantification of the luminescent signals. **H.** The overall survival of the two groups of mice treated with doxorubicin. (log-rank test, n = 12, p = 0.0124). *, p<0.01; **, p<0.05.

We then asked whether exogenous MSCs promote the proliferation and survival of tumors *in vivo*. Using a osteosarcoma mouse model with co-injection of MSCs with Saos-2, the bioluminescence imaging revealed that MSCs significantly enhanced the signal at the tumor site during the 4-week period (Figure 4A, 4C). Moreover, the tumor volume of the Saos-2 group that was injected with MSCs was larger than the control group (Saos-2, Figure 4B, 4D). In addition, co-injection with MSCs and Saos-2 cells significantly decreased the survival time of mice (Figure 4E). To evaluate the effectiveness of MSCs on chemotherapy, the mice were inoculated with Saos-2 or Saos-2/MSCs and treated with doxorubicin (10 mg/kg by weekly intraperitoneal injection (ip)). Only weak bioluminescence signals were observed in the Saos-2 group, while mice co-injected with MSCs had a stronger signal at week 4 (Figure 4F, 4G). Compared with the Saos-2 OS group, co-injection with MSCs significantly reduced the survival of the mice (Figure 4H).

### Inhibition of STAT3 enhances osteosarcoma cell sensitivity to chemotherapy *in vivo*

**Figure 5:**
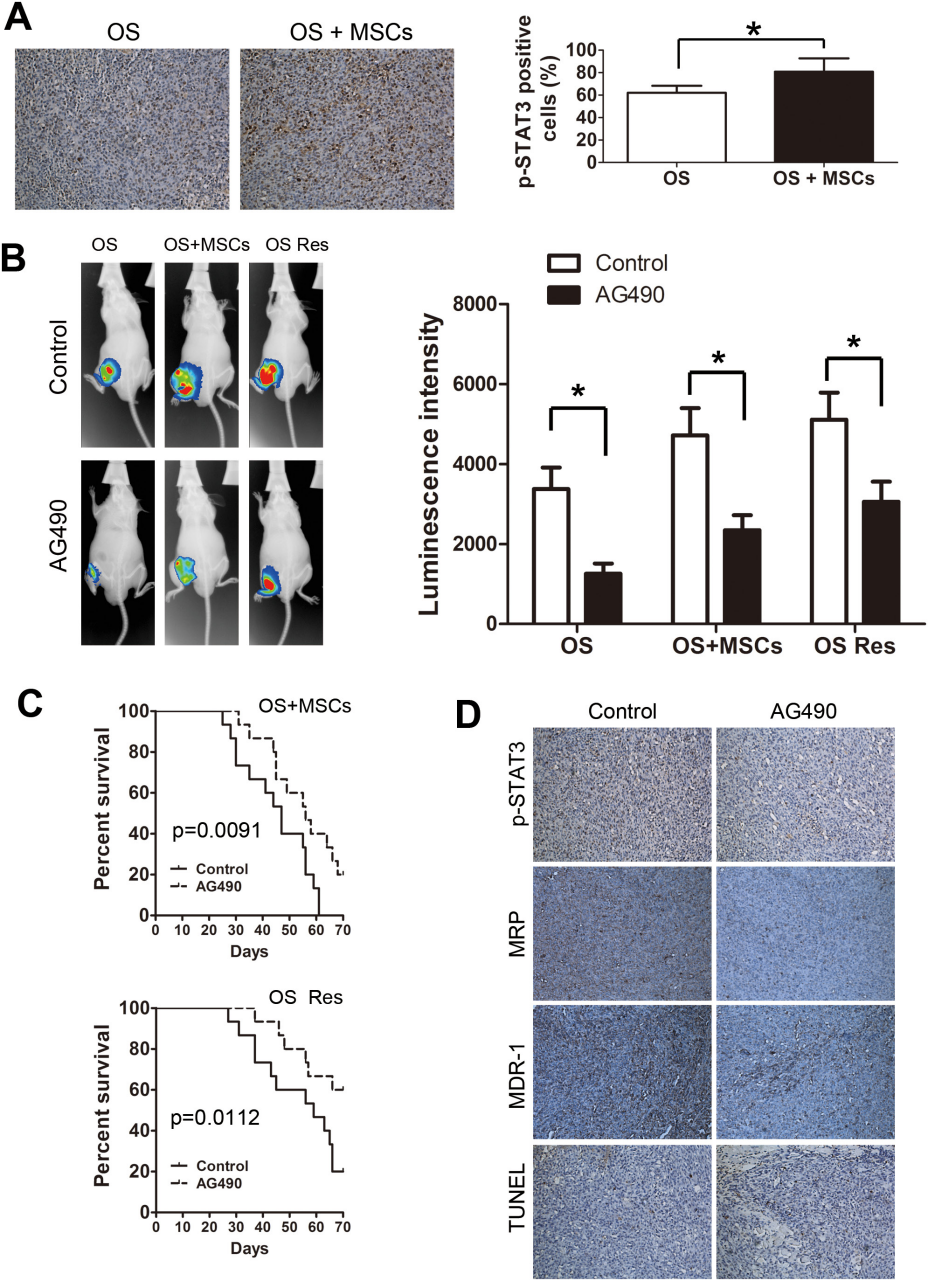
Inhibition of STAT3 enhances the sensitivity of osteosarcoma to chemotherapy *in vivo* **A.** Left, immunostaining of p-STAT3 was performed on tumors harvested from OS and OS+MSCs mice. Right, quantification of p-STAT3 positive-cells. **B.** Mice were injected with Saos-2+MSCs or doxorubicin-resistant Sao-2 cells and treated with doxorubicin and AG490 via intraperitoneal injection. Left, *in vivo* images of the mice at week 4. Right, quantification of the luminescent signals. **C.** Overall survival of the two group of mice that were treated with AG490. (log-rank test, n = 12. Top, OS+MSCs, p=0.0091; Bottom, Saos-2 Res, p=0.0112). **D.** The expression profiles of p-STAT3, MRP and MDR-1 protein levels were evaluated by immunohistochemistry assay. The level of apoptosis was examined *in vivo* by the TUNEL assay. *, p<0.01.

We next asked whether STAT3 signaling modulates chemotherapy effectiveness *in vivo*. The OS and OS+MSCs mice were sacrificed, and the STAT3 activation levels were detected by IHC. We observed that co-injection of tumor cells with MSCs dramatically increased the p-STAT3 levels in the tumor (Figure 5A). Then, mice were injected withSaos-2, Saos-2+MSCs or drug resistant Saos-2 cells (Saos-2 Res group) and treated weekly with doxorubicin and 500 μg AG490 (or DMSO in the control) via intraperitoneal injection for 4 weeks. While a strong signal was observed at the tumor sites in all three groups, injection with AG490 significantly decreased the bioluminescence intensity in these groups (Figure 5B). In addition, AG490 treatment in both Saos-2+MSCs and Saos-2 Res groups lengthened the survival times of the mice (Figure 5C). STAT3 activation increase expression of downstream pro-chemoresistant genes. Therefore we also evaluated the protein levels of MRP and MDR-1, which are known to promote chemotherapy resistance in tumors. The immunohistochemistry results showed that AG490 treatment decreased the activation of STAT3 and the expression of MRP and MDR-1. The TUNEL assay indicated an increase in cell apoptosis following the injection of AG490 (Figure 5D).

### STAT3 is critical for clinical chemotherapeutic outcomes in osteosarcoma

**Figure 6:**
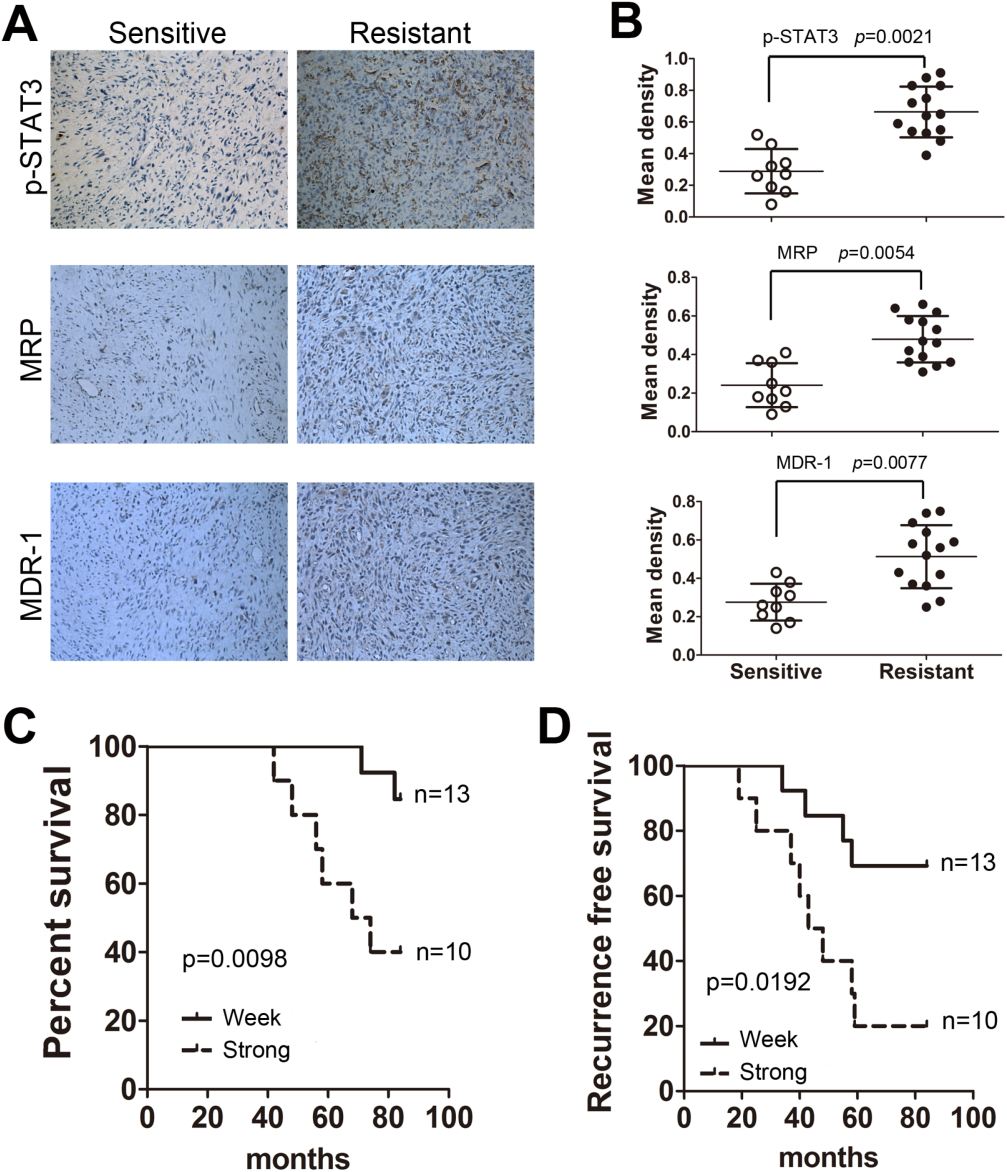
STAT3 is critical for the clinical chemotherapeutic outcomes of osteosarcoma Twenty-three patients (9 sensitive and 14 resistant) that received systematic chemotherapy and had their tumors resected by surgery were characterized for STAT3 markers. **A.** p-STAT3, MRP and MDR-1 expression levels were examined by immunohistochemistry assay. **B.** Quantification of the corresponding staining densities (mean±SD, t-test, p < 0.01). **C.** Correlation between the expression of p-STAT3 (staining < 0.5 and staining ≥0.5) and overall survival in osteosarcoma patients. **D.** Correlation between the expression of p-STAT3 (staining < 0.5 and staining ≥0.5) and recurrence-free survival in osteosarcoma patients.

To confirm a clinical role for STAT3 in osteosarcoma chemoresistance, tumors were collected during surgical resection from patients that were treated with systemic chemotherapy (9 sensitive and 14 resistant to chemotherapy, Supplementary Table S1). We observed that p-STAT3, MRP and MDR-1 expression levels were much higher in resistant tumors than sensitive samples (Figure 6A, 6B). Patients were divided according to the density of the p-STAT3 staining (more or less than 0.5) into weak (n=10) or strong groups (n=13). One sample from the resistant group showed a staining score less than 0.5. The survival analysis indicated that the outcome for patients in the p-STAT3 strong-staining group was significantly worse than the p-STAT3 weak-staining group (Figure 6C). In addition, the p-STAT3 weak-staining group showed a longer recurrence-free time after combined chemotherapy treatment and surgical resection (Figure 6D).

### MSC-induced resistance is mediated by IL-6 release

**Figure 7:**
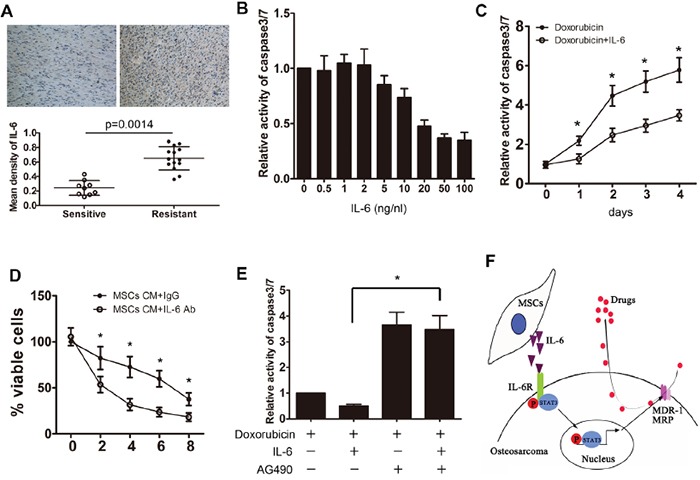
MSC-induced resistance is mediated by the release of IL-6 **A.** Chemotherapy sensitive and resistant osteosarcoma samples were collected, and the mean density of IL-6 was detected by immunohistochemistry. **B.** Saos-2 cells were exposed to 20 μg/ml doxorubicin and then treated with different concentrations of IL-6 for 48 h. The relative caspase 3/7 activity in the tumor cells was examined. **C.** Saos-2 cells were exposed to 20 μg/ml doxorubicin and then treated with 20 ng/ml of IL-6 for 4 days. The relative caspase 3/7 activity was examined daily. **D.** Saos-2 cells were cultured in the presence or absence of MSCs CM and then treated with IL-6 neutralizing antibody. The number of viable cells was then assessed using the CCK-8 assay. **E.** Saos-2 cells were exposed to 20 μg/ml doxorubicin and then treated with IL-6 and/or AG490. The caspase 3/7 activity was tested. **F.** Graphical depiction of the hypothetical pathway governing MSC-induced osteosarcoma resistance. Activation by IL-6 from MSCs may regulate drug resistance by modulating the expression of multiple drug resistant genes in osteosarcoma cells. n=3; *, p<0.01.

We previously demonstrated that IL-6, a major activator of STAT3 signaling, plays an important role in the interaction between MSCs and osteosarcoma cells. We found that tumor cells significantly promoted the IL-6 production in MSCs (Supplementary Figure S4). Then we examined the effects of MSC-produced IL-6 on osteosarcoma cells. The expression of IL-6 across the clinical samples was assessed by immunohistochemistry assay. We observed that the expression of IL-6 was higher in the resistant osteosarcoma samples as compared to the sensitive samples (Figure 7A). Then, Saos-2 cells that were exposed to different concentrations of IL-6 (ranging from 0-100 ng/ml) were treated with doxorubicin for 48 h. Our findings showed that treatment of Saos-2 cells with IL-6 (greater than 20 ng/ml) inhibited chemotherapy-induced caspase 3/7 activity significantly (Figure 7B). To confirm the protective effects of IL-6 on the tumor cells, Saos-2 cells were treated with doxorubicin in the presence or absence of 20 ng/ml IL-6 for 4 days. We showed that IL-6 treatment led to a decrease in caspase 3/7 activity in the tumor cells (Figure 7C). In addition, we found that blockade of IL-6 in MSC CM via specific neutralizing antibodies attenuated the protective effect of MSCs on Saos-2 tumor cells (Figure 7D). Finally, STAT3 inhibition by AG490 rescued the protective effect of IL-6 on drug-induce caspase 3/7 activation (Figure 7E).

## DISCUSSION

Our study identifies an important mechanism of chemotherapy resistance that is mediated by MSCs. We show that MSCs that reside in the osteosarcoma microenvironment confer resistance to multiple types of chemotherapy. We revealed that IL-6/STAT3 signaling is responsible for the induction of resistance by MSCs and drugs. MSC-driven chemotherapy resistance in osteosarcoma cells was confirmed *in vivo* using mouse models. Our examination of the interactions between osteosarcoma cells and MSCs sheds new light on the understanding of osteosarcoma chemotherapy sensitivity.

The tumor stroma, involving tumor–host cellular interactions, plays an important role in tumor growth, metastasis and survival [21, 22]. Activated fibroblasts in the tumor stroma are commonly referred to as carcinoma-associated fibroblasts (CAFs) or tumor associated fibroblasts (TAFs) [23]. Accumulating evidence suggests that CAFs/TAFs in the tumor microenvironment are derived from MSCs [24, 25]. Interestingly, osteosarcoma frequently occurs within the metaphy-seal area of long bones [26], which harbors major pools of MSCs. Here, we present novel data, which suggest that the protective effect of MSCs on osteosarcoma cell viability was primarily due to pro-survival versus pro-proliferative changes, which has not been much explored so far. Several previous studies have shown that MSCs have a pro-apoptotic effect on tumor cells. Sun et al. found that intravenous injection of MSC increased PARP-1 and caspase-3 cleavage in mammary carcinoma xenografts [27]. Our observation suggests that MSCs suppresses osteosarcoma cell death that is associated with serum depletion and chemotherapy. Our data clearly demonstrate that under stress conditions, such as low serum and drug treatment, MSCs promote survival, a role that is highly relevant to clinical chemotherapeutic interventions.

The use of co-injection of MSCs with Saos-2 osteosarcoma tumor cells in the tibia of mice represents a helpful model to examine the interaction between MSCs and tumor cells *in vivo*. However, we recognize that this model is limited in that MSCs were not recruited from the bone marrow by primary tumors. It is important to note that similar co-injection models have been used to examine other aspects of MSCs–tumor interaction *in vivo*. Karnoub et al. found that co-injection of human breast cancer cells with MSCs increased the metastasis of tumor cells [28]. Our current study raises the question of whether MSCs would play a similar protective role on apoptosis, as they are recruited from the bone marrow. Our observations clearly demonstrate that the protective effect of MSCs on osteosarcoma cells is specific. These artificially enriched MSCs in tumors developed stromal elements with functional properties that are similar to TAFs [25, 29]. Indeed, a protective effect against drug-induced apoptosis has been reported for tumor-associated macrophages in breast cancer [30].

Constitutive activation of STAT3 has been observed in many cancers and has been linked to increased cell proliferation and survival [31]. Many studies have further demonstrated that aberrant activation of STAT3 directly confers a drug-resistance phenotype [32]. Interestingly, the inhibition of STAT3 signaling has been shown to downregulate the expression of survival proteins and restores the sensitivity of cells to certain drugs [33]. Here, we showed that MSCs activate STAT3 signaling in osteosarcoma cells both *in vitro* and *in vivo*. We further showed that the use of a STAT3 inhibitor significantly enhanced the chemotherapeutic efficacy and prolonged the survival of tumor-bearing mice. Using a drug-resistant osteosarcoma cell line, we showed that MSCs-induced STAT3 activation was similar to drug-induced STAT3 activation and that inhibition of STAT3 in both mice tumor models decreased tumor growth and prolonged overall survival. Our previous research suggests that IL-6 may contribute to the survival of osteosarcoma cells. In line with this, we demonstrated in this study that MSC-produced IL-6 exerted a protective effect on the drug-induced apoptosis. However, inhibition of STAT3 by AG490 increased the sensitivity of IL-6-treated tumor cells to doxorubicin. Although the STAT3 activation could be induced by different pathways, our findings highlight that STAT3 inhibition could prevent both MSCs and drug-induced chemoresistance.

In osteosarcoma, overexpression of MDR-1 has been considered a risk factor for adjuvant chemotherapy [34]. Work by Susa et al. has shown that inhibition MDR-1 greatly improved the sensitivity of osteosarcoma to drugs [35]. We have found that co-injection of MSCs with Saos-2 cells increased the expression of MDR-1 and MRP, suggesting that MSCs may enhance the expression of these proteins through activation of STAT3. Furthermore, our clinical samples showed increased expression of p-STAT3, MRP and MDR-1 in chemoresistant tumors, suggesting that the STAT3-associated genes may be relevant to poor outcomes of osteosarcoma chemotherapy.

In summary, our findings reveal a systemic mechanism of resistance via the activation of STAT3 by IL-6 from MSCs, with the subsequent up-regulation of MRP and MDR-1 expression. It is especially notable that osteosarcoma cells that express relatively high levels of STAT3, either activated by drugs or induced by MSCs in its environment, were significantly sensitized to the apoptotic effects of drugs by STAT3 inhibition. Considering that MSCs are indispensable for tissue regeneration and may be difficult to completely ablate from the organism, our findings introduce important players to the field of chemotherapy resistance and indicate that the STAT3 pathway may be a druggable pathway to restore environment-induced chemoresistance.

## MATERIALS AND METHODS

### Cells and reagents

The human osteosarcoma cell lines Saos-2 and U2-OS were purchased from the Chinese Academy of Sciences (Shanghai, China). Luciferase-labeled Saos-2 cells were generated in our lab as previously described [19]. To generate a drug-resistant cell line, Saos-2 cells were treated with 1 μg/mL doxorubicin or cisplatin, and the medium was refreshed to eliminate dead cells twice a week. Cells were treated until no cell death was detected. Human bone marrow-derived MSCs were obtained from the proximal femur during orthopedic surgery as previously described [36] and processed according to the ethical guidelines of the Shanghai Ninth People's Hospital, Shanghai, China. MSCs that were passaged 3 times were used in our experiments. All of the cells were cultured in Dulbecco's Modified Eagle Medium (DMEM) containing 10% fetal bovine serum (FBS) and supplemented with 1% penicillin–streptomycin.

Reagents: The doxorubicin and cisplatin used were purchased from Sigma, MO, USA, and AG490 was purchased from Calbiochem, CA, USA.

### Patients and specimens

Twenty-three patients who underwent surgical resection for their primary osteosarcoma lesion between January 2006 and December 2012 were evaluated in this study. The median age of the patients was 27 years (range, 15–68 years). This study obtained approval from the ethic committee of Ninth People's Hospital of Shanghai Jiao Tong University School of Medicine, and written informed consent was obtained from the patients or their legal guardians. Then, according to the inhibition rate of doxorubicin in the tumor susceptibility test results, the patient's histological specimens were divided into sensitive or resistant groups (Supplementary Table S1) [37]. No significant difference was observed in composition regarding age or sex in these two groups (p > 0. 05).

### MSCs conditioned medium

MSCs were plated in 10-cm dishes and grown to a confluent monolayer. Cells were rinsed with phosphate-buffered saline (PBS) and cultured in serum-free DMEM for 24 h. The conditioned media was then collected, centrifuged at 1,000 × g for 10 min, and filtered through a 0.22-μm filter (Millipore, Billerica, MA).

### Apoptosis assay

For the Annexin V assay, sub-confluent cells were cultured in 6-well plates and harvested by trypsinization. Cells were washed twice with cold PBS and then resuspended in binding buffer. Annexin V and propidium iodide (PI) staining were performed according to the manufacturer's instructions (Invitrogen, Carlsbad, CA, USA), and the Annexin V and PI signals were measured by FACS. To detect caspase 3/7 activities, the cells were cultured in 96-well plates at a density of 1×10^4^ per well. After 24 h of treatment, the caspase 3/7 activity was determined by a Caspase-Glo kit (Promega). All of the experiments were performed in triplicate.

### Cell viability analysis

Saos-2 cells were seeded in 96-well plates at a density of 1×10^4^ per well and treated as follows: the culture medium was discarded, and the cells were rinsed 3 times with PBS. Subsequently, the viable cells were quantitated using a cell counting kit-8 (CCK-8, Dojindo, Japan) according to the manufacturer's instructions. Briefly, the cells were incubated in DMEM medium containing 10 μl of CCK-8 solution at 37°C for 2.5 hours. Then, the optical density (OD) at 450 nm was determined using a microplate reader (BIOTEK, Vermont, USA), and the ratio of viable cells was calculated.

### Western blot

The cells were washed in ice-cold PBS before lysis with cell lysis buffer (Cell Signaling Technology, MA, USA). All samples were clarified by centrifugation and the protein concentrations were determined using the BCA Protein Assay (Thermo Scientific, IL, USA). Equal amounts of total protein lysates were separated through SDS-PAGE and transferred to a nitrocellulose membrane. The membranes were blotted with antibodies against STAT3, p-STAT3, or β-actin (Cell Signaling Technology, MA, USA). Bound antibodies were detected with an Odyssey Infrared Imaging System (LI-COR Biosciences, Lincoln, NE, USA). Densitometric analysis of the protein bands was performed with an image-pro plus 4.5 software (Media Cybernetics, Silver Spring, MD).

### Animals and xenograft model

Studies in mice were performed using a protocol approved by the Animal Ethics Committee of the Shanghai Jiaotong University School of Medicine. Four-week-old male BALB/c nude mice were injected with 5×10^6^ luciferase-labeled Saos-2 cells or co-injected with 5×10^6^ MSCs into the left proximal tibia. For the drug treatment, the mice received 10 mg/kg of doxorubicin via intraperitoneal injection (ip) weekly. For STAT3 inhibition, the mice were treated weekly with an intraperitoneal injection of DMSO (control) or 500 μg AG490 (AG490 group). The luminescence activity in the mice was monitored weekly using an *In Vivo* Imaging System (IVIS, Xenogen, Alameda, CA). The tumor volume was measured every 3 days until the animals were sacrificed. The mice were sacrificed at week 4, and in situ tumor samples were collected for histological analysis. For the survival assay, 12 mice per group were maintained until death to allow for the calculation of survival curves.

### Histology

Tumor samples from the nude mice or clinical resections were fixed overnight in 10% neutral-buffered formalin. The tissues were embedded in paraffin and cut into 5-μm sections. The slides were incubated at 60°C for 30 min, deparaffinized in xylene and rehydrated through a graded ethanol series. After antigen retrieval, intrinsic peroxidase activity was blocked by incubation with 3% hydrogen peroxide for 10 min. The slides were covered with the appropriately diluted primary antibodies and incubated at 4°C overnight. After three washes in TBS-T for 5 min each, secondary antibodies were applied for 1 h, and the staining was developed using the Dako Cytomation Envision staining kit according to the manufacturer's instructions. The level of apoptosis was assessed with a terminal deoxynucleotidyltransferase–mediated dUTP nick end labeling kit (TUNEL, Roche Applied Science) according to the manufacturer's instructions.

### Statistical analyses

The data are represented as the means ± standard deviations (SD). Comparisons between groups were performed using Student's t-test, and one-way ANOVA was used for multiple comparisons. Survival rates were compared using Kaplan–Meier survival curves. Statistical significance was set at *p* < 0.05.

## SUPPLEMENTARY TABLE AND FIGURES


