# Supplementary material for: Mesenchymal stem cells promote osteosarcoma cell survival and drug resistance through activation of STAT3
Source: Oncotarget. 2016 Jun 22;7(30):48296–308. doi: 10.18632/oncotarget.10219 (PMC5217018; doi:10.18632/oncotarget.10219)
Supplement: Supplementary file 1 [file oncotarget-07-48296-s001.pdf]

# Mesenchymal stem cells promote osteosarcoma cell survival and drug resistance through activation of STAT3

## SUPPLEMENTARY TABLE AND FIGURES

**Supplementary Table S1: Drugs sensitivity was tested by ATP tumor chemosensitivity assay (ATP-TCA)**

| Patients | | Sex | Age | IC50(%) | IC90(%) |
|---|---|---|---|---|---|
| Sensitive | **1** | F | 15 | 5.6 | 88.9 |
| | **2** | F | 17 | 20.6 | 149.7 |
| | **3** | M | 20 | 13.5 | 133.5 |
| | **4** | M | 23 | 30.9 | 89.3 |
| | **5** | M | 24 | 23.5 | 128.5 |
| | **6** | F | 38 | 4.8 | 77.4 |
| | **7** | F | 51 | 10.8 | 95.3 |
| | **8** | M | 58 | 14.4 | 57.7 |
| | **9** | M | 64 | 9.3 | 54.39 |
| Resistant | **1** | M | 15 | 70.6 | 179.5 |
| | **2** | M | 16 | 37.6 | 145.3 |
| | **3** | F | 19 | 57.4 | 141.2 |
| | **4** | M | 22 | 28.5 | 138.7 |
| | **5** | M | 23 | 39.2 | 115.1 |
| | **6** | F | 27 | 65.3 | 125.2 |
| | **7** | F | 27 | 34.6 | 162.7 |
| | **8** | M | 36 | 46.4 | 119.5 |
| | **9** | M | 53 | 50.3 | 130.6 |
| | **10** | M | 60 | 28.37 | 105.7 |
| | **11** | F | 63 | 36.5 | 143.2 |
| | **12** | F | 63 | 41.2 | 162.1 |
| | **13** | F | 66 | 26.3 | 109.2 |
| | **14** | M | 68 | 37.3 | 127.2 |

Tumor specimens (1-2 $cm^3$) were taken during primary surgery. The chemosensitivity was determined by the ATP-TCA kit (DCS Innovative Diagnostik Systeme, Hamburg, Germany) according to the manufacture's instructions. Sensitive: IC50<25% TDC or IC90<100% TDC; Resistant: IC50>25% test drug concentration (TDC) and IC90>100% TDC.

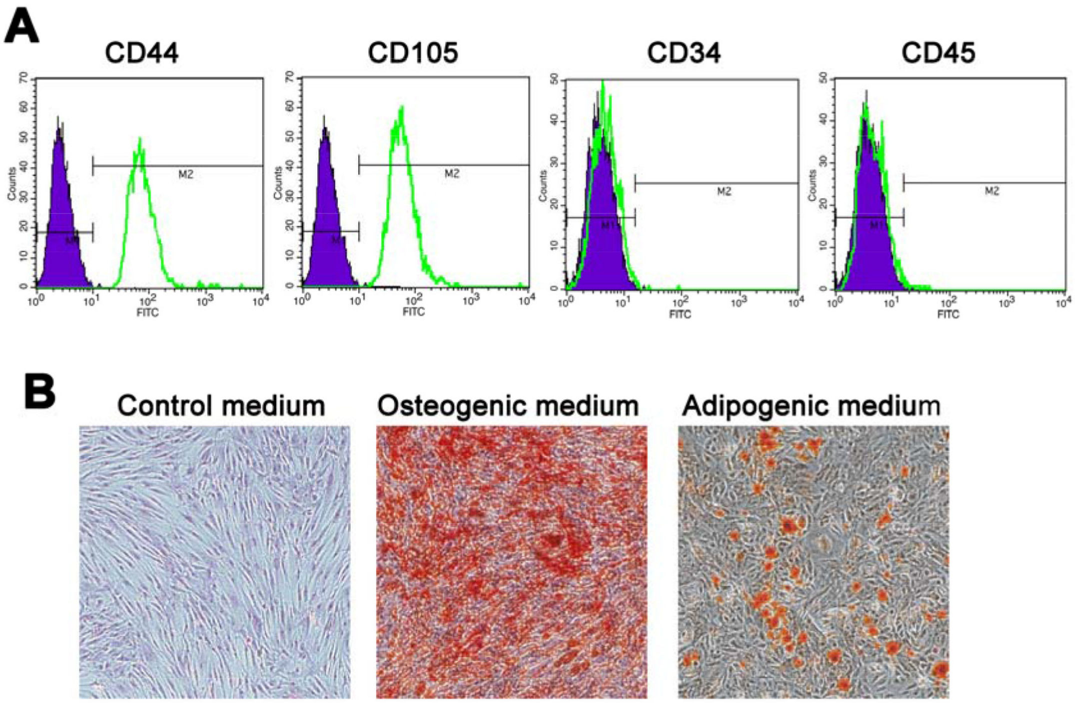


**Supplementary Figure S1: Characterization of MSCs. A.** Expression of cell surface markers in MSCs. The filled histogram curves represents the autofluorescent control. The open histogram curves show the staining with respective primary and secondary antibodies. **B.** Left: undifferentiated MSCs; Middle: Osteogenic differentiation of MSCs staining with Alizarin Red S at week 2; Right:, Adipogenic differentiation of MSCs staining with Oil Red O at week 2.

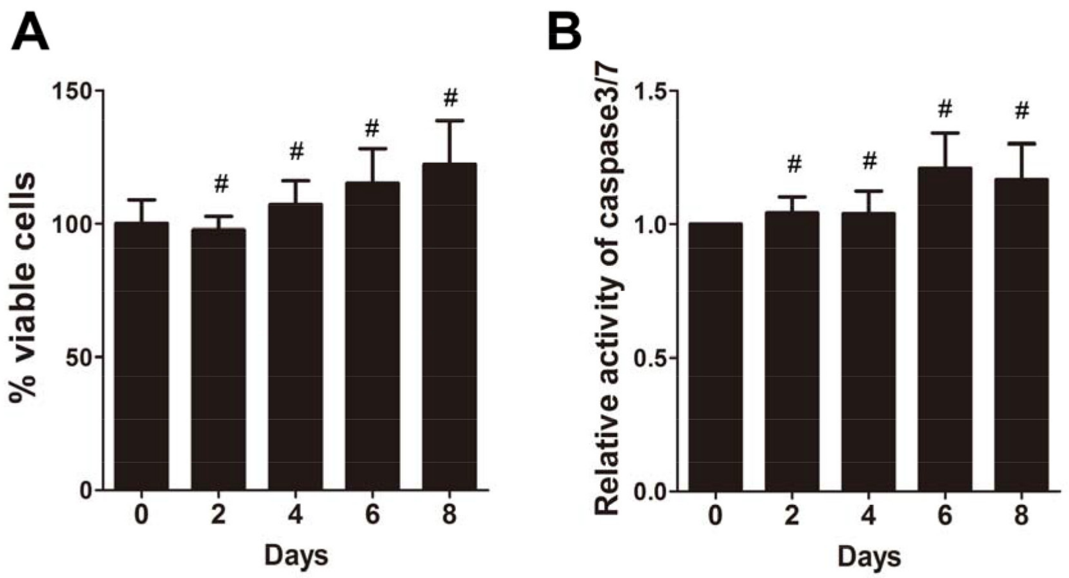


**Supplementary Figure S2: 1% FBS has no effect on the proliferation and apoptosis of tumor.** MSCs were cultured in DMEM containing 1% FBS for 8 days. **A.** Cell proliferation was evaluated by the CCK-8 assay. **B.** caspase 3/7 activity was examined at the indicated times. #, $p>0.05$.

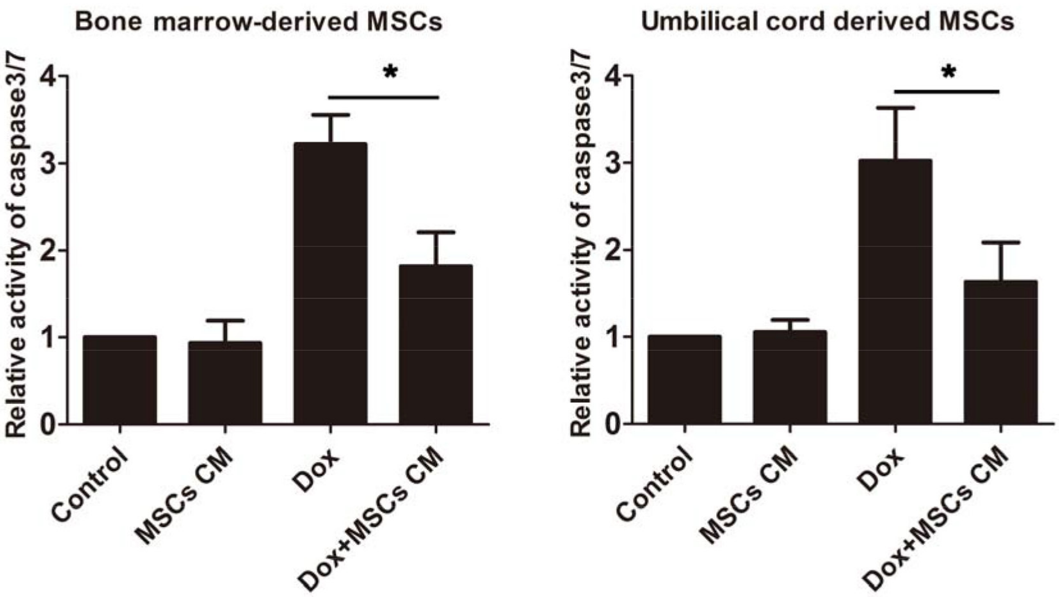


**Supplementary Figure S3: MSCs from different tissues protect Saos-2 cells from doxorubicin-induced apoptosis.** Saos-2 cells were treated with MSCs CM and/or doxorubicin for 48h, caspase 3/7 activity in the cells was detected. Left: Bone marrow-derived MSCs CM. Right: Umbilical cord derived MSCs CM. The human umbilical cord derived MSCs were purchased from Chinese Academy of Sciences, Shanghai, China. *, $p<0.05$.

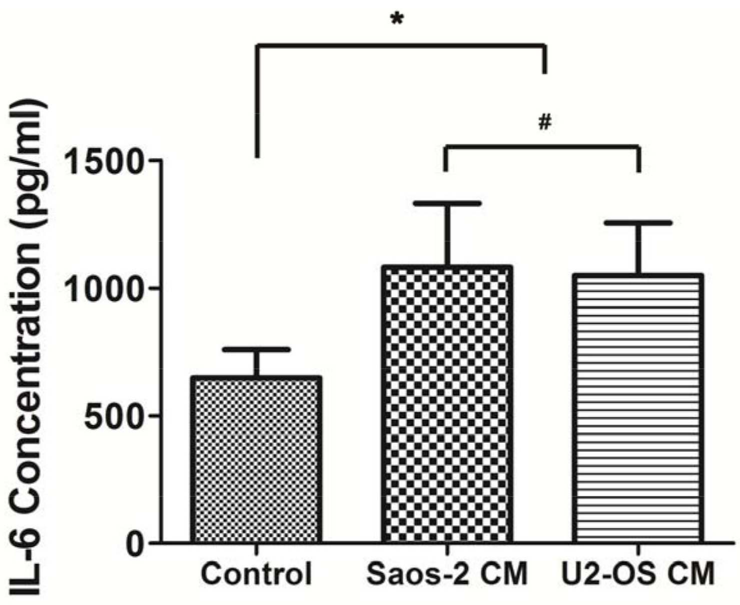


**Supplementary Figure S4: The osteosarcoma cells promote the IL-6 expression in MSCs.** MSCs were incubated in Saos-2 CM or U2-OS CM for 48h. Then the medium was replaced with DMEM without FBS and cultured for another 24h. The IL-6 concentration in the MSCs supernatant was detected by ELISA. *, $p<0.05$; #, $p>0.05$.
